# Complete response of a giant metastatic neck mass in tonsillar squamous cell carcinoma achieved with radiotherapy combined with immunotherapy: a case report

**DOI:** 10.3389/fonc.2026.1816432

**Published:** 2026-05-08

**Authors:** Wenqin Huang, Jian Wang, Xiaohua Hong, Gang Peng

**Affiliations:** 1 Cancer Center, Union Hospital, Tongji Medical College, Huazhong University of Science and Technology, Wuhan, Hubei, China; 2 Breast Cancer Center, Hubei Cancer Hospital, Tongji Medical College, Huazhong University of Science and Technology, National Key Clinical Specialty Construction Discipline, Hubei Provincial Clinical Research Center for Breast Cancer, Wuhan Clinical Research Center for Breast Cancer, Wuhan, Hubei, China; 3 Hubei Key Laboratory of Precision Radiation Oncology, Wuhan, Hubei, China; 4 Institute of Radiation Oncology, Union Hospital, Tongji Medical College, Huazhong University of Science and Technology, Wuhan, Hubei, China

**Keywords:** complete response, giant metastatic lymph node, immunotherapy, radiotherapy, tonsillar squamous cell carcinoma

## Abstract

Tonsillar squamous cell carcinoma (SCCT) is generally sensitive to chemoradiotherapy; however, treating advanced, resistant lesions remains challenging. This report presents a case of a 57-year-old male with SCCT. At initial diagnosis in May 2022, only small metastatic lymph nodes were observed in right neck levels II and III. The patient declined surgery and radiotherapy. From June to August 2022, he received 4 cycles of albumin-bound paclitaxel + cisplatin chemotherapy combined with tislelizumab, followed by tislelizumab plus capecitabine treatment from September 2022 to May 2023. Treatment was voluntarily interrupted from June 2023 to March 2024 due to intolerance, totaling approximately 18 months. By November 2024, the right neck mass had progressed into a giant, fused lesion measuring 12.27 × 7.85 cm. Subsequently, radiotherapy to the tonsillar primary bed (GTV 60 Gy in 20 fractions) combined with camrelizumab was administered. After 20 fractions, clinical examination and imaging confirmed near-total resolution of the mass, with good skin healing, achieving a complete response and leaving only mild skin fibrosis. As of April 15, 2025, there was no evidence of local recurrence or distant metastasis. This case suggests that in a heavily pretreated patient with traditionally treatment-resistant, rapidly progressing SCCT and an ultra-bulky metastatic mass, the combination of radiotherapy and immunotherapy was associated with a complete response. However, given the multiple prior lines of systemic therapy, the contribution of immune priming or delayed immunotherapeutic effects cannot be excluded. Nevertheless, this observation highlights a potential synergistic opportunity for similar advanced patients.

## Introduction

Squamous cell carcinoma of the tonsil (SCCT) accounts for approximately 23% of oropharyngeal cancers (1), and its incidence has risen significantly due to human papillomavirus (HPV) infection (1). While early-stage SCCT responds well to standard treatments such as surgery and radiotherapy (2), the management of advanced cases, particularly those with large tumors or extensive lymph node metastasis, poses significant challenges, with studies indicating that 5-year local control rates may fall below 60% (3). Radiotherapy serves as a cornerstone in the management of SCCT (4), especially for patients who are unsuitable for surgery or require organ function preservation (4). For advanced SCCT, the standard radiotherapy dose typically ranges from 60 to 70 Gy (5); however, controlling large tumors remains difficult, with reported 5-year local control rates of approximately 61% for T4 and 78% for T3 disease (5). In recent years, the combination of immunotherapy with radiotherapy has shown potential (6), possibly by enhancing immune responses to improve therapeutic efficacy (7). This case report presents a patient with SCCT who developed a giant metastatic cervical lymph node following initial treatment failure and achieved complete tumor resolution through radiotherapy combined with immunotherapy (7), suggesting the significant value of this combined strategy for resistant, advanced SCCT.

## Case description

A 57-year-old male presented with a painless right neck mass. Physical examination revealed a firm, poorly mobile mass in right neck levels II and III, initially measuring approximately 10mm × 10mm, with no palpable lymphadenopathy on the left. Ultrasound-guided core needle biopsy suggested infiltrating or metastatic squamous cell carcinoma, with no definitive lymph node structure visible microscopically. Immunohistochemistry showed: P40 positive, P16 positive, Ki67 proliferation index ~60%, EBER negative, CK5/6 positive, CK7 partially positive, GATA-3 negative, CK20 negative, PAX8 negative (**Figure 1**). PET-CT revealed multiple enlarged, fused lymph nodes in right levels II/III with increased metabolism (SUVmax 8.5), consistent with malignancy; the right tonsil was slightly larger than the contralateral side with higher metabolism (SUVmax 12.9 vs. 5.1 contralaterally) (**Figure 2**). On physical examination of the oropharynx, the right tonsil appeared enlarged with an exophytic, papillary lesion. Endoscopic visualization at the time of biopsy confirmed a papillary, exophytic tumor involving the right tonsil, consistent with the subsequent pathological findings. The patient subsequently underwent a right tonsil biopsy, with pathology indicating: (Right tonsil) squamous cell carcinoma, showing mainly papillary, exophytic growth patterns.

**Figure 1:**
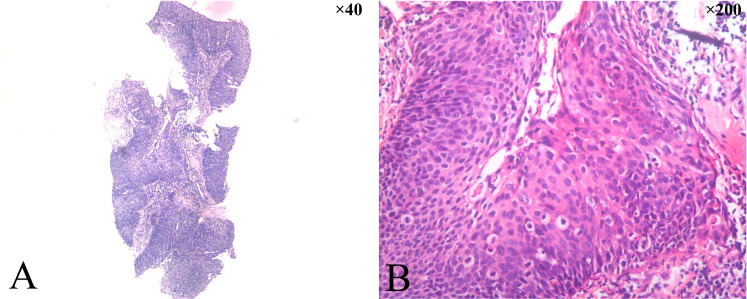
Histopathological findings of the patient’s right neck metastatic lymph node. **(A, B)** H&E staining of the right neck enlarged lymph node biopsy specimen is consistent with infiltrating or metastatic squamous cell carcinoma.

**Figure 2:**
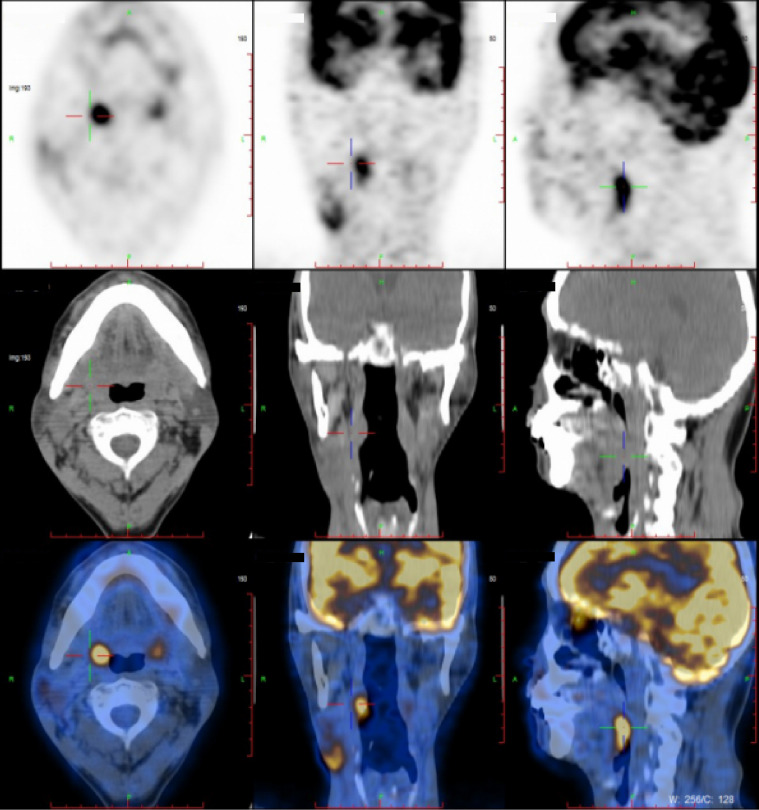
PET-CT findings of the patient. PET-CT imaging shows: Multiple fused lymph nodes in levels II/III with focal necrosis and active metabolism (SUVmax=8.5), consistent with metastatic squamous carcinoma; the right tonsil is enlarged compared to the contralateral side with significantly higher metabolism (SUVmax=12.9 vs. 5.1 contralaterally), highly suggestive of a primary malignancy; no definite malignant lesions were detected in other surveyed areas.

At initial presentation, the patient denied any associated symptoms such as odynophagia, dysphagia, hoarseness, or otalgia. His past medical history included type 2 diabetes mellitus for 5 years (managed with insulin), hyperuricemia (treated with colchicine), and a history of schistosomiasis-related liver disease. He had a 35-year smoking history of 40 cigarettes per day and a 35-year alcohol consumption history of approximately 50 mL per week, with alcohol cessation 3 months before the initial diagnosis. His family history was significant for diabetes mellitus in his mother, one brother, one sister, and one elder brother; his father was healthy.

The diagnosis of tonsillar malignancy T4N2M0 was established according to AJCC 9^th^ edition. Based on immunohistochemistry and PET-CT findings, it was considered HPV-positive oropharyngeal squamous cell carcinoma (OPSCC). As the patient was initially diagnosed in May 2022, when the AJCC 8th edition was the standard in clinical practice, we also present the staging according to the 8th edition: for HPV-positive OPSCC, the T4N2M0 classification corresponds to Stage III.

At initial diagnosis, the patient declined surgery and radiotherapy. He therefore received 4 cycles of albumin-bound paclitaxel (260 mg/m² on day 1), cisplatin (75 mg/m² on day 1), and tislelizumab (200 mg on day 1), administered every 3 weeks from June to August 2022 (specific dates: June 2, June 24, July 17, and August 8, 2022). Magnetic resonance imaging (MRI) during this period showed fused right neck lymph nodes, maximum size approximately 20 × 12 mm, indicating progression. Subsequently, from September 2022 to May 2023, he received tislelizumab (200 mg every 3 weeks) for approximately 8 cycles, along with metronomic capecitabine (at a dose of approximately 625 mg twice daily) given continuously. From June 2023 to March 2024, the patient chose to interrupt treatment due to intolerance to adverse effects like vomiting. On August 5, 2024, MRI and ultrasound revealed continued enlargement of the right neck lymph nodes, measuring 65 × 45 × 56 mm.

On August 22, 2024, the patient was enrolled in a clinical trial titled: “A Study of BGB-R046 as Monotherapy and in Combination with Tislelizumab in Solid Tumors” (ClinicalTrials.gov Identifier: NCT06487858). The patient participated in the phase 1a part of this trial, receiving BGB-R046 monotherapy. BGB-R046 is an IL-15 (interleukin-15) pro-drug primarily acting on IL-15 receptors on immune cells, thereby activating immune effector cells such as NK cells and CD8+ T cells to enhance anti-tumor immune responses (8, 9). The patient withdrew from the trial in September 2024 due to neck mass progression. On September 6 and 25, 2024, he received 2 cycles of gemcitabine (1000 mg/m² on days 1 and 8 of a 3-week cycle), cisplatin (75 mg/m² on day 1), and toripalimab (240 mg on day 1), with a 3-week interval between cycles. He discontinued this regimen voluntarily due to poor tolerance. By November 18, 2024, the mass had progressed to 12.27 × 7.85 cm, with multiple epidermal lesions, ulcers, bleeding, and exudate on the surface of the giant mass (**Figure 3A**).

**Figure 3:**
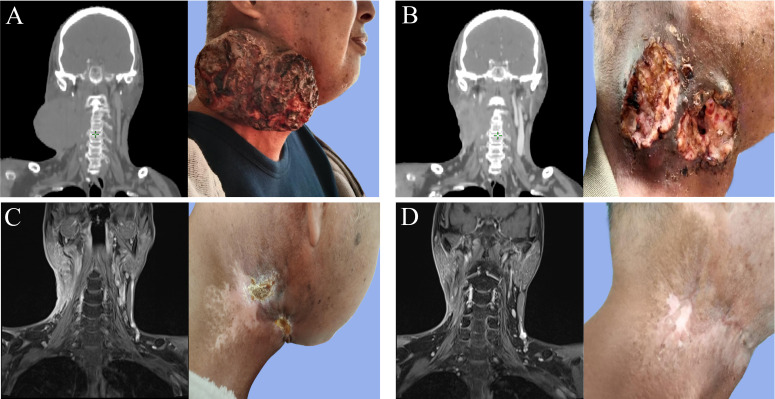
CT and MRI findings of the patient. **(A)** Neck CT simulation image from November 22, 2024 (pre-radiotherapy) showing the giant right neck mass measuring 12.27 × 7.85 cm, alongside a photograph of the mass for comparison. **(B)** Neck CT image from December 13, 2024 (pre-field modification) showing the reduced size of the right neck mass, alongside a corresponding photograph. **(C)** Contrast-enhanced MRI of the neck and oropharynx from January 3, 2025 (one week post-radiotherapy) indicating significant reduction of both the right neck mass and the right palatine tonsil compared to previous scans, alongside a photograph. **(D)** Neck contrast-enhanced MRI from July 15, 2025 (7 months post completion of radiotherapy combined with immunotherapy) showing the right neck mass has markedly reduced, is ill-defined, with a small enhancing nodule approximately 7 mm in diameter remaining. Oropharyngeal contrast-enhanced MRI indicates the right palatine tonsil is similar in size to the previous examination.

Starting November 26, 2024, the patient received volumetric modulated arc therapy (VMAT) to the tonsillar primary bed. The treatment regimen referenced the SCART (Stereotactic Centralized Ablative Radiation Therapy) concept, using a hypofractionated schedule of 60 Gy in 20 fractions (3 Gy per fraction) (10). Although this dose level is close to that used in palliative radiotherapy, it was delivered with curative intent given the patient’s poor tolerance to standard chemoradiotherapy and the urgent need for rapid tumor debulking. The gross tumor volume (GTV) included the primary right tonsillar tumor and the metastatic right neck lymph nodes (the giant mass). The planning target volume (PTV) was generated by adding a 3–5 mm isotropic margin to the GTV to account for setup uncertainties and organ motion. After significant tumor shrinkage was observed on the verification CT (December 13, 2024), the treatment fields were modified. This field modification is a typical example of adaptive radiotherapy, ensuring that the target volume was re-optimized to spare normal tissues while maintaining adequate dose coverage. The isodose distributions of the initial and adapted plans are shown in **Figures 4A, B**, respectively. Concurrent camrelizumab (200 mg) was administered on day 1 of radiotherapy, which constituted one cycle given the 4-week radiotherapy course; no additional camrelizumab cycles were given during radiotherapy. During radiotherapy, the right neck mass gradually shrank (**Figure 3B**). Upon completion, visual inspection showed complete disappearance of the right neck mass, with local skin almost completely healed. MRI re-examination on January 13, 2025, showed an irregular patchy enhancement in the right neck measuring approximately 55 × 26 mm, and the right palatine tonsil was significantly smaller than before (**Figure 3C**). Follow-up until July 15, 2025, with neck contrast-enhanced CT and MRI, revealed disappearance of the neck mass, leaving only a small enhancing nodule about 7 mm in diameter, and the right palatine tonsil was similar to previous examinations (**Figure 3D**). Tumor response was evaluated according to RECIST version 1.1. The target lesion (the giant neck mass) was no longer measurable, and the residual 7 mm enhancing nodule had a short axis of <10 mm, which meets the RECIST criteria for complete response of non-target lesions (i.e., complete resolution). No new lesions appeared. Therefore, the overall response was classified as a complete response (CR). No PET-CT was performed at this time point to assess metabolic response, which is acknowledged as a limitation. The chronological summary of diagnostic assessments, therapeutic interventions, and outcomes is presented in **Table 1**.

**Figure 4:**
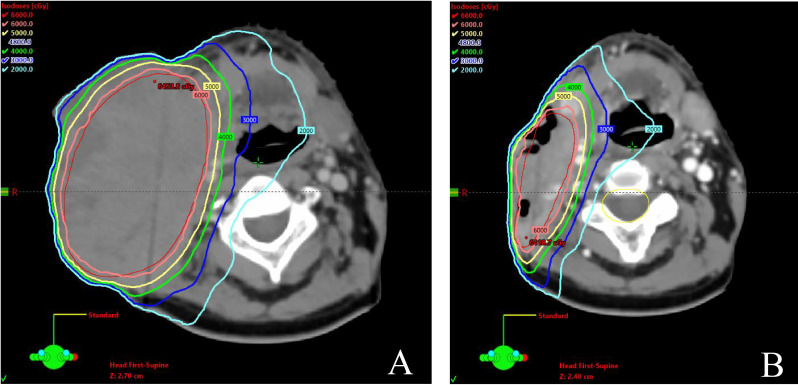
Isodose distribution of the initial and adapted VMAT plans. **(A)** Initial VMAT plan for the giant right neck mass and tonsillar primary bed. Isodose lines (cGy): 6600 (red), 6000 (orange), 5000 (yellow), 4800 (light green), 4000 (dark green), 3000 (blue), 2000 (purple). The prescribed dose was 60 Gy in 20 fractions (3 Gy per fraction). Maximum point dose: 6453.8 cGy. **(B)** Adapted VMAT plan after tumor shrinkage (field modification). Maximum point dose: 6418.7 cGy. The spinal cord and other organs at risk were kept within tolerance.

**Table 1:** Chronological summary of diagnostic assessments, therapeutic interventions, and outcomes.

Date	Event	Key details
May 2022	Initial diagnosis	57-year-old male; right neck mass (approx. 10×10 mm); right tonsil biopsy confirmed SCCT; PET-CT showed right tonsil (SUVmax 12.9) and right neck nodes (SUVmax 8.5); staging: T4N2M0, AJCC 9th (Stage III per AJCC 8th for HPV-positive OPSCC); patient declined surgery and radiotherapy.
June-Aug 2022	First-line: chemotherapy + tislelizumab	4 cycles of albumin-bound paclitaxel (260 mg/m²) + cisplatin (75 mg/m²) + tislelizumab (200 mg) every 3 weeks (June 2, June 24, July 17, Aug 8). MRI showed progression: right neck nodes enlarged to ~20×12 mm.
Sep 2022-May 2023	Second-line: tislelizumab + capecitabine	Tislelizumab (200 mg every 3 weeks) for approx. 8 cycles, along with continuous daily metronomic capecitabine (approx. 625 mg twice daily).
June 2023-Mar 2024	Treatment interruption	Patient voluntarily interrupted treatment due to intolerance (vomiting).
Aug 5, 2024	Progression assessment	MRI/ultrasound: right neck nodes enlarged to 65×45×56 mm.
Aug-Sep 2024	Clinical trial: BGB-R046 monotherapy	Enrolled in NCT06487858, phase 1a, received BGB-R046 (IL-15 prodrug). Withdrew due to neck mass progression.
Sep 2024	Third-line: gemcitabine + cisplatin + toripalimab	2 cycles (Sep 6 and 25, 2024): gemcitabine (1000 mg/m² days 1 and 8) + cisplatin (75 mg/m² day 1) + toripalimab (240 mg day 1), every 3 weeks. Discontinued due to poor tolerance.
Nov 18, 2024	Pre-radiotherapy assessment	Neck mass progressed to 12.27 × 7.85 cm with epidermal lesions, ulcers, bleeding, and exudate.
Nov 26, 2024-Dec 2024 (approx. 4 weeks)	Fourth-line: radiotherapy + camrelizumab	VMAT to tonsillar primary bed: GTV 60 Gy in 20 fractions (3 Gy/fraction), PTV 6020 cGy, concurrent with camrelizumab (200 mg day 1 of radiotherapy). Neck mass gradually shrank during treatment.
Jan 13, 2025	Post-radiotherapy assessment (1 week post-RT)	MRI: irregular patchy enhancement in right neck (approx. 55×26 mm); right palatine tonsil significantly smaller.
July 15, 2025	Follow-up (7 months post-RT)	Neck contrast-enhanced CT/MRI: neck mass resolved, residual 7 mm enhancing nodule (short axis <10 mm); right palatine tonsil similar to prior exam.
April 15, 2025 (latest follow-up)	Outcome	No evidence of local recurrence or distant metastasis; overall response: CR (complete response) per RECIST 1.1.

SCCT, squamous cell carcinoma of the tonsil; OPSCC, oropharyngeal squamous cell carcinoma; HPV, human papillomavirus; MRI, magnetic resonance imaging; PET-CT, positron emission tomography-computed tomography; VMAT, volumetric modulated arc therapy; GTV, gross tumor volume; PTV, planning gross tumor volume; CR, complete response; RECIST, Response Evaluation Criteria in Solid Tumors.

This table corresponds to Item 5 (Timeline) of the CARE guidelines checklist. The timeline may also be presented as a standalone figure. According to the CARE guidelines, the timeline “presents relevant events in the patient’s history in chronological order in a figure or table and offers a succinct summary of one or more key events in the case, enabling the reader to readily view core elements of the case report”.

## Discussion

This case report describes a 57-year-old male patient diagnosed with HPV-positive tonsillar squamous cell carcinoma. After multiple treatment failures, the neck mass progressed into a giant, fused lesion measuring 12.27 × 7.85 cm. Complete response was achieved with VMAT to the tonsillar primary bed. The prescribed dose was 60 Gy in 20 fractions (3 Gy daily) to both GTV and PTV, delivered with reference to the SCART concept and using an adaptive strategy after early tumor shrinkage, concurrent with camrelizumab immunotherapy. The neck mass completely resolved, leaving only mild skin fibrosis. Follow-up until April 15, 2025, showed no evidence of recurrence or metastasis.

Before interpreting the response as solely due to the radiotherapy-immunotherapy combination, it is important to acknowledge the patient’s extensive treatment history. He received four cycles of chemotherapy plus tislelizumab, followed by tislelizumab plus capecitabine, then BGB-R046 (an IL-15 prodrug) monotherapy, and finally two cycles of gemcitabine, cisplatin, and toripalimab. Although the tumor progressed through all these regimens, prior immunotherapy and IL-15 agonist may have primed the immune system or induced a delayed immunotherapeutic effect. For instance, tislelizumab (anti-PD-1) could have expanded tumor-specific T cell clones, and BGB-R046 might have activated NK and CD8+ T cells, even without objective tumor regression at the time. The subsequent radiotherapy, by inducing immunogenic cell death and releasing tumor antigens, could have acted as the “final trigger” that converted a subclinical immune-primed state into a dramatic clinical response. Thus, the complete response observed likely reflects a cumulative effect of sequential immune modulation rather than the exclusive action of the final combination.

HPV-positive oropharyngeal squamous cell carcinomas, such as SCCT, are often considered suitable for treatment de-escalation strategies due to their generally better prognosis compared to HPV-negative cases (2). However, this patient’s disease exhibited aggressive behavior with rapid progression despite multiple systemic therapies, highlighting the heterogeneity of HPV-positive OPSCC and the necessity for personalized treatment. Standard treatment for advanced OPSCC typically involves concurrent chemoradiotherapy with radiation doses of 66–70 Gy (11). Recent de-escalation studies for low-risk HPV-positive patients suggest that 60 Gy radiotherapy can maintain similar progression-free survival rates (12). However, the regimen used in this case—60 Gy in 20 fractions (3 Gy per fraction)—differs from standard definitive radiotherapy for SCCT, which typically delivers 66–70 Gy in 33–35 fractions (1.8-2.0 Gy per fraction) (11). From a radiobiological perspective, using an α/β ratio of 10 Gy for head and neck squamous cell carcinoma, the biologically effective dose (BED) of 60 Gy/20F is approximately 78 Gy (BED = total dose × [1 + dose per fraction/10]), compared with 79.2 Gy for 66 Gy/33F and 84 Gy for 70 Gy/35F. Thus, the BED of this hypofractionated regimen is within the range of conventional schedules, albeit slightly lower than the highest standard doses. The larger fraction size (3 Gy) may theoretically increase the risk of late normal tissue toxicity, but the patient tolerated treatment well with only mild skin fibrosis. The choice of this regimen was influenced by several clinical considerations: (1) the tumor was rapidly progressing through multiple prior therapies and had reached a giant size (12.27 × 7.85 cm), necessitating a shorter overall treatment time (4 weeks vs. 6–7 weeks) to achieve timely cytoreduction and relieve symptoms; (2) the patient had poor tolerance to previous treatments, and a shorter course was preferred to improve compliance; (3) emerging evidence suggests that hypofractionated radiotherapy may enhance immunogenic cell death and tumor antigen release, potentially providing a stronger synergistic effect with concurrent immunotherapy (13). While the independent contribution of this fractionation scheme to tumor control cannot be isolated from the concurrent camrelizumab, the rapid shrinkage observed during radiotherapy suggests that the radiation itself played a significant role. Nonetheless, we emphasize that this regimen remains exploratory, and its safety and efficacy require validation in controlled studies.

The key aspect of this case lies in the combination of radiotherapy and immunotherapy. Immune checkpoint inhibitors (ICIs) are approved for use in recurrent or metastatic head and neck squamous cell carcinoma (R/M HNSCC) (6). Radiotherapy can induce immunogenic cell death, releasing tumor antigens and enhancing anti-tumor immune responses, thereby creating a synergistic effect with immunotherapy (13). To provide a comprehensive overview of the current evidence for this combination in HNSCC, we summarize below the major studies across different treatment settings, including prospective trials, retrospective analyses, and case reports (**Table 2**).

**Table 2:** Summary of selected studies on radiotherapy combined with immunotherapy for head and neck squamous cell carcinoma.

Study (year)	Study type	Setting	RT dose/fractionation	Immunotherapy agent	Sequencing	Key findings
Prospective/clinical trials						
NeoRTPC02 trial (2025) (14)	Phase II single-arm	Neoadjuvant, locally advanced resectable HNSCC (stage III-IVB)	Low-dose RT (details in original)	Tislelizumab (anti-PD-1) + chemotherapy	Induction (pre-surgery)	pCR rate 60.9%, MPR 21.7%, R0 resection 100%; manageable toxicity
Liu et al. (2025) (15)	Phase II single-arm (preliminary)	Neoadjuvant, locally advanced resectable HNSCC	Low-dose RT (1Gy × 4 fractions)	Cardonilizumab (anti-PD-1/CTLA-4 bispecific) + chemotherapy	Induction (pre-surgery)	pCR 33.3%, MPR 66.7%, ORR 100%; all R0 resections
NRG-HN004 (completed, terminated early) (16)	Phase II/III RCT	Locoregionally advanced HNC (cisplatin-ineligible)	Standard fractionation RT (concurrent)	Durvalumab (anti-PD-L1)	Concurrent	Testing RT+durvalumab vs RT+cetuximab; phase III terminated due to futility?
NCT03829722 (2024 completed) (17)	Phase II single-arm	High-risk HPV-related OPSCC	Fractionated RT (70Gy) + carboplatin/paclitaxel	Nivolumab (anti-PD-1)	Priming, concurrent, adjuvant	2-year PFS 65% in high-risk p16+ OPSCC
NCT03799445 (completed) (18)	Phase II	Advanced HPV-positive OPSCC	Reduced-field IMRT	Ipilimumab + Nivolumab	Induction → concurrent	Evaluating safety, CR rate, and 2-year PFS
NCT03952585 (NRG-HN005) (ongoing) (19)	Phase II/III RCT	Early-stage, HPV-positive, non-smoking OPSCC	Reduced-dose RT	Nivolumab	Concurrent	Testing reduced-dose RT+nivolumab vs RT+cisplatin
KEYSTROKE trial (NCT03546582) (ongoing) (20)	Phase II RCT	Locoregionally recurrent or second primary HNSCC	SBRT re-irradiation	Pembrolizumab	SBRT → pembrolizumab (or SBRT alone)	Testing SBRT+pembrolizumab vs SBRT alone for PFS
NBTXR3 trial (NCT03589339) (phase I) (21)	Phase I dose escalation	R/M HNSCC (anti-PD-1 naïve or resistant)	SBRT + NBTXR3 (radioenhancer)	Nivolumab or Pembrolizumab	Sequential	Evaluating safety and feasibility in ICI-resistant patients
Carbon ion RT + ICI trial (ongoing) (22)	Prospective	R/M HNSCC, melanoma, NSCLC, urothelial	Carbon ion radiotherapy	Pembrolizumab/Atezolizumab/Nivolumab	Concurrent	Evaluating ORR in palliative setting
Retrospective studies						
Cheng et al. (2025) (23)	Retrospective	R/M HNSCC	Various (real-world)	Various ICIs	Variable	RT group DCR 94.6% vs 61.8% without RT; RT improved outcomes
Quad Shot study (2024) (24)	Retrospective (n=70)	Symptomatic R/M HNC (palliative)	Quad Shot (3.7Gy bid ×4 fractions, cycles q3-4w)	Pembrolizumab/Nivolumab/Cemiplimab	Concurrent	12-month local control: 84.7% (RT+ICI) vs 63.3% (RT alone); P = 0.038
Case reports/case series						
Choi et al. (2019) (25)	Case series (n=2) + literature review	R/M HNSCC	SBRT	Various ICIs	Sequential	Complete response via abscopal effect; sustained responses
Forner et al. (2020) (26)	Case report + review	R/M HNSCC	RT	Nivolumab	Sequential	Abscopal effect observed
Case report with ipilimumab+nivolumab (2019) (27)	Case report	mHNSCC	Palliative RT (Quad Shot)	Ipilimumab + Nivolumab	Sequential	Abscopal effect with sustained response
Present case	Case report	HPV-positive SCCT with giant metastatic neck mass (12.27 × 7.85 cm)	Hypofractionated VMAT (60 Gy/20 fractions, 3 Gy/fraction)	Camrelizumab (anti-PD-1)	Concurrent	Complete response per RECIST 1.1; mild skin fibrosis only

RT, radiotherapy; ICI, immune checkpoint inhibitor; HNSCC, head and neck squamous cell carcinoma; HNC, head and neck cancer; OPSCC, oropharyngeal squamous cell carcinoma; SCCT, squamous cell carcinoma of the tonsil; R/M, recurrent/metastatic; pCR, pathological complete response; MPR, major pathological response; ORR, objective response rate; DCR, disease control rate; PFS, progression-free survival; SBRT, stereotactic body radiation therapy; VMAT, volumetric modulated arc therapy; GTV, gross tumor volume; CR, complete response; RCT, randomized controlled trial.

In the neoadjuvant setting, the phase II NeoRTPC02 trial (NCT05343325) investigated low-dose radiotherapy combined with tislelizumab, albumin-bound paclitaxel, and cisplatin in 28 patients with untreated stage III-IVB resectable HNSCC, achieving a pathological complete response (pCR) rate of 60.9% and a major pathological response (MPR) rate of 21.7%, with all surgically treated patients achieving R0 resection (14). In the definitive concurrent setting for high-risk HPV-positive oropharyngeal cancer, a phase II trial (NCT03829722) evaluated fractionated radiotherapy with carboplatin/paclitaxel plus nivolumab, with 2-year PFS as the primary endpoint (17). The ongoing NRG-HN005 trial (NCT03952585) is a phase II/III study comparing reduced-dose radiotherapy plus nivolumab versus standard-dose radiotherapy plus cisplatin in early-stage, non-smoking-associated HPV-positive oropharyngeal cancer, aiming to assess whether de-intensification with immunotherapy can maintain efficacy while reducing toxicity (19). Conversely, the phase II/III NRG-HN004 trial (NCT03258554) compared radiotherapy with concurrent durvalumab versus radiotherapy with cetuximab in cisplatin-ineligible patients with locoregionally advanced HNSCC; the trial was terminated early due to futility, with 2-year PFS of 50.6% in the durvalumab group versus 63.7% in the cetuximab group (HR 1.33), suggesting that durvalumab did not improve outcomes compared to cetuximab in this patient population (16).

In the recurrent/metastatic and palliative settings, the retrospective Quad Shot study (2024) analyzed 70 patients with R/M HNC treated with hypofractionated Quad Shot radiotherapy (3.7 Gy twice daily × 4 fractions, repeated every 3–4 weeks) with or without concurrent ICIs; the 12-month local control rate was 84.7% in the RT+ICI group versus 63.3% in the RT alone group (P = 0.038), demonstrating that the addition of ICIs significantly improved local control in the palliative setting (24). The ongoing KEYSTROKE trial (NCT03546582) is a randomized phase II study evaluating SBRT re-irradiation with or without pembrolizumab for locoregionally recurrent or second primary HNSCC, with progression-free survival as the primary endpoint (20). Additionally, the phase I Study 1100 (NCT03589339) evaluated NBTXR3, a novel radioenhancer, administered as intratumoral injection followed by SBRT in combination with nivolumab or pembrolizumab in patients with anti-PD-1 naïve or resistant HNSCC; preliminary results demonstrated objective responses in 25% of ICI-resistant patients and 54% of ICI-naïve patients, with disease control rates of 75% and 77%, respectively, suggesting that NBTXR3/RT may help overcome ICI resistance (21). The phase II ICONIC trial (NCT05229614) is prospectively evaluating the addition of carbon ion radiotherapy to pembrolizumab in patients with advanced solid tumors (including HNSCC) who have achieved disease stability on immunotherapy, with objective response rate as the primary endpoint, exploring the potential immunomodulatory effects of high-LET radiation (22). Finally, several case reports have documented abscopal effects following radiotherapy combined with ICIs in R/M HNSCC, including responses with SBRT and nivolumab, as well as with the Quad Shot regimen combined with ipilimumab and nivolumab (25, 27).

Beyond these general observations, the unique biology of HPV-positive OPSCC may further potentiate the radiotherapy-immunotherapy synergy. HPV-driven tumors are highly immunogenic, characterized by elevated baseline levels of tumor-infiltrating lymphocytes (especially CD8+ T cells), upregulation of PD-L1, and the expression of viral oncoproteins E6 and E7, which serve as strong neoantigens (2, 28). These features are associated with improved responses to immune checkpoint inhibitors compared to HPV-negative disease (29). Radiotherapy can augment this immunogenicity by inducing immunogenic cell death, releasing viral and tumor-associated antigens, and upregulating MHC class I expression on tumor cells (13, 30). In HPV-positive models, radiation has been shown to enhance cross-presentation of E6/E7 antigens, leading to stronger systemic antitumor immunity when combined with PD-1/PD-L1 blockade (31). Hypofractionated regimens, such as the 3 Gy per fraction used here, may be particularly effective at triggering immunogenic cell death and promoting dendritic cell activation (32). In this patient, the complete response of a giant, previously progressive mass may therefore reflect not only direct cytoreduction but also a reignition of HPV-specific immune responses that had been primed by prior immunotherapies (tislelizumab and the IL-15 agonist). This interpretation aligns with the concept of “radio-immunotherapy” converting an immune-resistant lesion into a T-cell-inflamed tumor, especially in HPV-positive cancers with pre-existing immune infiltrates.

It is worth noting that PD-L1 expression and CPS were not assessed in this patient at any time point. In the current clinical practice for recurrent or metastatic head and neck squamous cell carcinoma (HNSCC), immune checkpoint inhibitors (ICIs) such as nivolumab and pembrolizumab have demonstrated survival benefits regardless of PD-L1 expression levels in the second-line or later-line setting (6). For HPV-positive oropharyngeal cancers, which are highly immunogenic, the response to ICIs may be even less dependent on PD-L1 expression (2, 28). Therefore, the decision to administer tislelizumab, toripalimab, and camrelizumab in this heavily pretreated patient with progressive disease was based on clinical necessity and the lack of effective alternative options, rather than on a specific PD-L1 threshold. The absence of PD-L1 testing does not invalidate the observed clinical response, but it does limit direct comparison with biomarker-driven studies.

The complete response in this case, while not proof of a novel strategy given the confounding prior therapies, supports the potential value of combining radiotherapy with immunotherapy in resistant, giant lesions, and suggests that such a combination may be considered as part of a salvage regimen—particularly in patients with prior immune exposure.

However, this case report has limitations. Treatment occurred within a clinical trial context; the applicability of the 60 Gy dose and fractionation schedule requires further validation. The patient’s prior exposure to multiple systemic therapies may have influenced the tumor’s response to subsequent treatment. Furthermore, the follow-up period is relatively short; longer-term follow-up is needed to assess response durability and late toxicities. Additionally, while we classified the response as complete according to RECIST 1.1 based on morphological imaging, the absence of metabolic imaging (e.g., PET-CT) at the time of response assessment precludes confirmation of a complete metabolic response. The small residual enhancing nodule (7 mm) could represent post-treatment fibrosis or inflammation rather than viable tumor, but longer follow-up is required to confirm its benign nature. Future research should focus on optimizing radiotherapy dose/fractionation, exploring optimal immunotherapy combinations, and validating this strategy in advanced SCCT through randomized controlled trials.

## Conclusion

This case demonstrates that in a heavily pretreated, treatment-resistant HPV-positive SCCT with a giant neck mass, the combination of radiotherapy and camrelizumab was associated with a complete response. However, the potential roles of immune priming and delayed effects from prior immunotherapies (including anti-PD-1 and IL-15 agonist) must be considered. The non-conventional regimen of 60 Gy in 20 fractions combined with camrelizumab shows promise, but its broader applicability requires validation in controlled studies. This case underscores the need for further research to disentangle the contributions of sequential immune modulations and to optimize the timing of radiotherapy in the course of immunotherapy for advanced SCCT. This case emphasizes the importance of personalized treatment in complex cases and calls for more clinical trials to explore optimal strategies for combining radiotherapy and immunotherapy to improve outcomes for patients with advanced SCCT.

## Data Availability

The original contributions presented in the study are included in the article/supplementary material. Further inquiries can be directed to the corresponding authors.
